# A randomized controlled trial of a shared decision making intervention for diabetes prevention for women with a history of gestational diabetes mellitus: The Gestational diabetes Risk Attenuation for New Diabetes (GRAND study)

**DOI:** 10.1016/j.cct.2022.107007

**Published:** 2022-11-13

**Authors:** Ruth Madievsky, Amanda Vu, Felicia Cheng, Janet Chon, Norman Turk, Ashley Krueger, Jacob Krong, Richard Maranon, Sandra Liu, Christina S. Han, Keith C. Norris, Carol Mangione, Jessica Page, Samuel Thomas, O. Kenrik Duru, Tannaz Moin

**Affiliations:** a Department of Medicine, Division of General Internal Medicine-Health Services Research, University of California, 1100 Glendon Ave STE 850, Los Angeles, CA 90024, USA; b Healthcare Delivery Institute, Office of Research, Intermountain Healthcare, 5026 S. State St, Murray, UT 84107, USA; c Division of Maternal-Fetal Medicine, Department of Obstetrics and Gynecology, University of California, 200 Medical Plaza, Suite 430, Los Angeles, CA 90095, USA; d Department of Medicine, Division of General Internal Medicine-Health Services Research, University of California, David Geffen School of Medicine at UCLA, 1100 Glendon Ave STE 850, Los Angeles, CA 90024, USA; e Department of Maternal-Fetal Medicine, Intermountain Healthcare. Department of Maternal-Fetal Medicine, University of Utah Health, 8th Ave & C St E, Salt Lake City, UT 84143, USA; f Department of Internal Medicine, Intermountain Healthcare, 5121 Cottonwood St, Murray, UT 84017, USA; g HSR&D Center for the Study of Healthcare Innovation, Implementation & Policy, VA Greater Los Angeles Healthcare System, Los Angeles, CA 90073, USA; h Department of Medicine, Division of Endocrinology, Diabetes & Metabolism, UCLA, Los Angeles, CA 90024, USA; i Jonathan and Karin Fielding School of Public Health, University of California, Los Angeles, CA, USA

**Keywords:** Gestational diabetes, Type 2 diabetes, Shared decision making, Decision aid, Prediabetes

## Abstract

**Background:**

Gestational diabetes mellitus (GDM) is a risk factor for the development of type 2 diabetes. Metformin and lifestyle change through a Diabetes Prevention Program (DPP) are equally effective in preventing diabetes in patients with a GDM history, so women can choose a strategy based on their preferences. This study aims to test whether shared decision making (SDM) can help women with a history of GDM increase adoption of evidence-based strategies and lose weight to lower their risk of incident diabetes in real-world settings.

**Methods:**

This pragmatic randomized controlled trial (RCT) will test the effectiveness of SDM for diabetes prevention among 310 overweight/obese women with a history of GDM and prediabetes from two large health care systems (*n* = 155 from UCLA Health and n = 155 from Intermountain Healthcare). The primary outcome is the proportion of participants who lose ≥5% body weight at 12 months. Secondary outcomes include uptake of DPP and/or metformin and other patient-reported outcomes such as patient activation and health-related quality of life. Rates of GDM in a subsequent pregnancy will be an exploratory outcome. A descriptive analysis of costs related to SDM implementation will also be conducted.

**Conclusion:**

This is the first RCT to examine the effectiveness of SDM on weight loss, lifestyle change and/or metformin use, and other patient-reported outcomes in participants with a GDM history at risk of developing diabetes.

**Trial registration:**

ClinicalTrials.gov, NCT03766256.

## 1. Introduction

Gestational Diabetes Mellitus (GDM) is glucose intolerance first detected or diagnosed during pregnancy [1,2]. A history of GDM is the single strongest predictor of incident type 2 diabetes (T2DM), increasing the lifetime risk sevenfold [3,4]. Diabetes is associated with an increased risk of cardiovascular disease, kidney and eye disease, and nerve damage, among other serious sequelae [5]. Half of women with a history of GDM develop T2DM within 5 to 7 years of their GDM diagnosis, and up to 70% develop T2DM within 20 years [6]. Thus, engaging women with a history of GDM in evidence-based strategies for T2DM prevention is a critical public health and societal goal.

There is strong evidence from the Diabetes Prevention Program (DPP) trial supporting the efficacy of lifestyle change or metformin use among women with a GDM history since either option reduces incident T2DM by about 50% [7–9]. Among women with a similar degree of impaired glucose tolerance randomized to placebo (no lifestyle change or metformin) in this trial, a history of GDM increased T2DM incidence by 71% compared to no GDM history. The DPP Outcomes Study (DPPOS) also showed that treating 7 women with a history of GDM with metformin or 11 women with intensive lifestyle intervention (ILI) would prevent one case of T2DM over ten years [10]. ILI is the preferred option for diabetes prevention due to the additional health benefits beyond diabetes prevention, but real-world uptake of either ILI or metformin remains low [11–14]. At least 14 studies have adapted ILI for women with a history of GDM with moderate success [15–28], but few published reports have described the use of metformin for women in this population.

Since women with a history of GDM face a “preference-sensitive” decision with two evidence-based alternatives to prevent T2DM [29], SDM is an ideal approach to help them make a decision aligned with their values. SDM makes use of decision aid (DA) tools to help make the treatment decisions explicit, describe the available options with equipoise, and help patients and clinicians work together to make an informed decision [30]. This framework is particularly appropriate for women with a history of GDM. Evidence suggests most of these women recognize GDM as a proven risk factor for T2DM, but few believe they are personally at high risk [31]. Many women with a history of GDM report high levels of stress, fatigue, cultural role limitations, and time and financial constraints which can make uptake of and adherence to lifestyle interventions challenging [32–35]. Finally, many are disconnected from the health care system with limited interaction with health providers and few opportunities for recommended post-partum screening. In light of the need for clear, bidirectional communication about lifestyle change and metformin between providers and women with a history of GDM, the lack of studies evaluating SDM in diabetes prevention in this population is an important gap that must be addressed.

This is the first study testing the effectiveness of SDM for diabetes prevention in women with a history of GDM.

## 2. Methods

### 2.1. Study design

This multicenter randomized controlled trial (RCT) tests whether SDM can increase weight loss and adoption of evidence-based strategies to lower the risk of incident T2DM in women with a history of GDM and prediabetes at high risk of developing T2DM. Participants will be randomized to the SDM intervention versus control and the primary outcome will be the proportion of participants with ≥5% weight loss at 12 months (Table 1). Secondary outcomes include the proportion with ≥5% weight loss at 24 months, uptake of an evidence-based diabetes prevention strategy within 6 months, patient-reported outcomes (perception of diabetes risk, self-efficacy, patient activation, health-related quality of life) and annual follow-up screening for progression to T2DM (Table 1). We will also measure rates of GDM in a subsequent pregnancy as an exploratory, hypothesis-generating outcome, and we will conduct a descriptive analysis of costs related to SDM implementation.

### 2.2. Study setting

This RCT will take place within two large health care systems with locations across California, Utah, Nebraska, and Idaho.

### 2.3. Eligibility

**Fig. 1.**
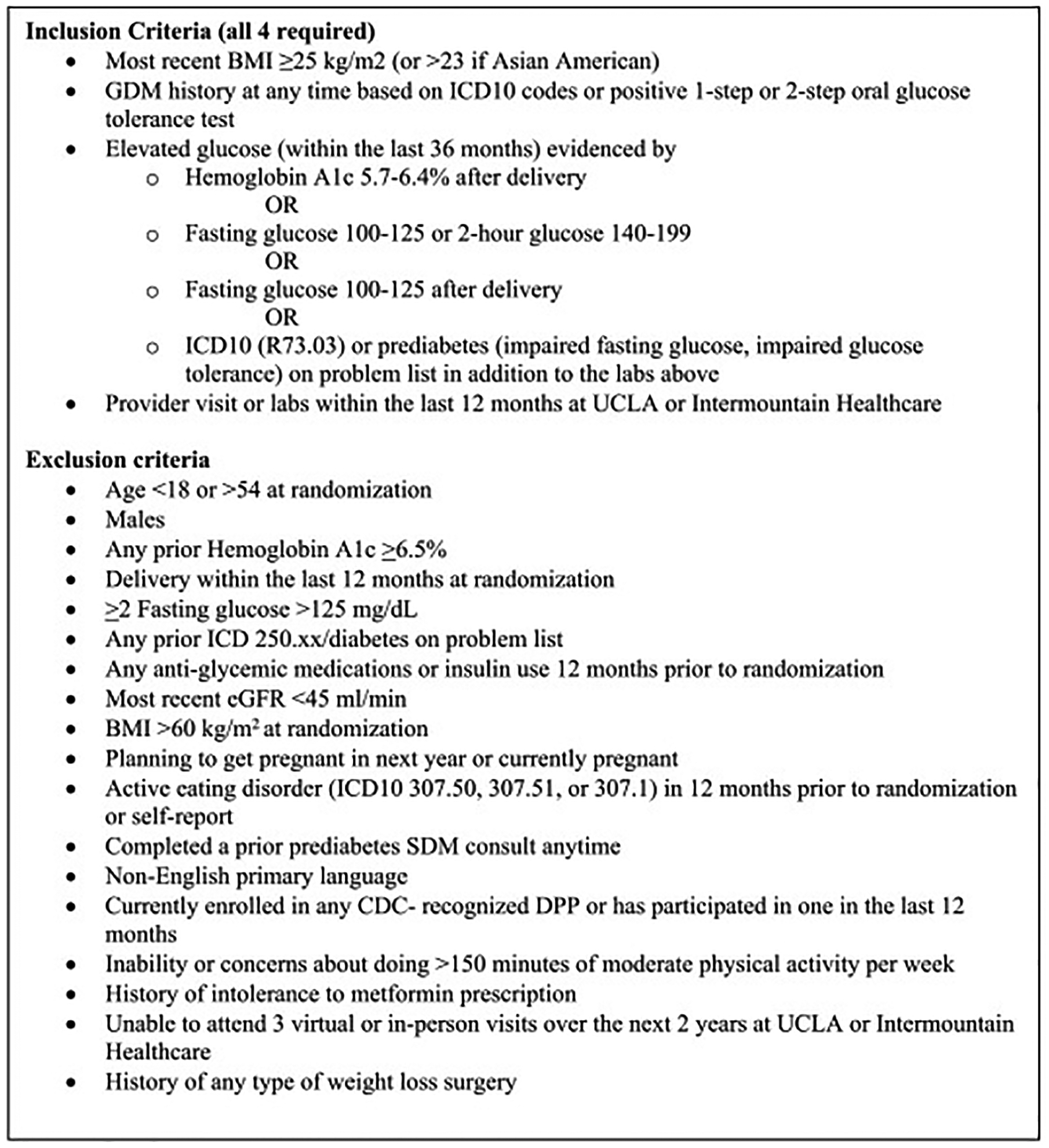
Eligibility criteria.

Our eligibility criteria are summarized in Fig. 1.

### 2.4. Participant identification and recruitment

#### 2.4.1. Randomization

**Fig. 2.**
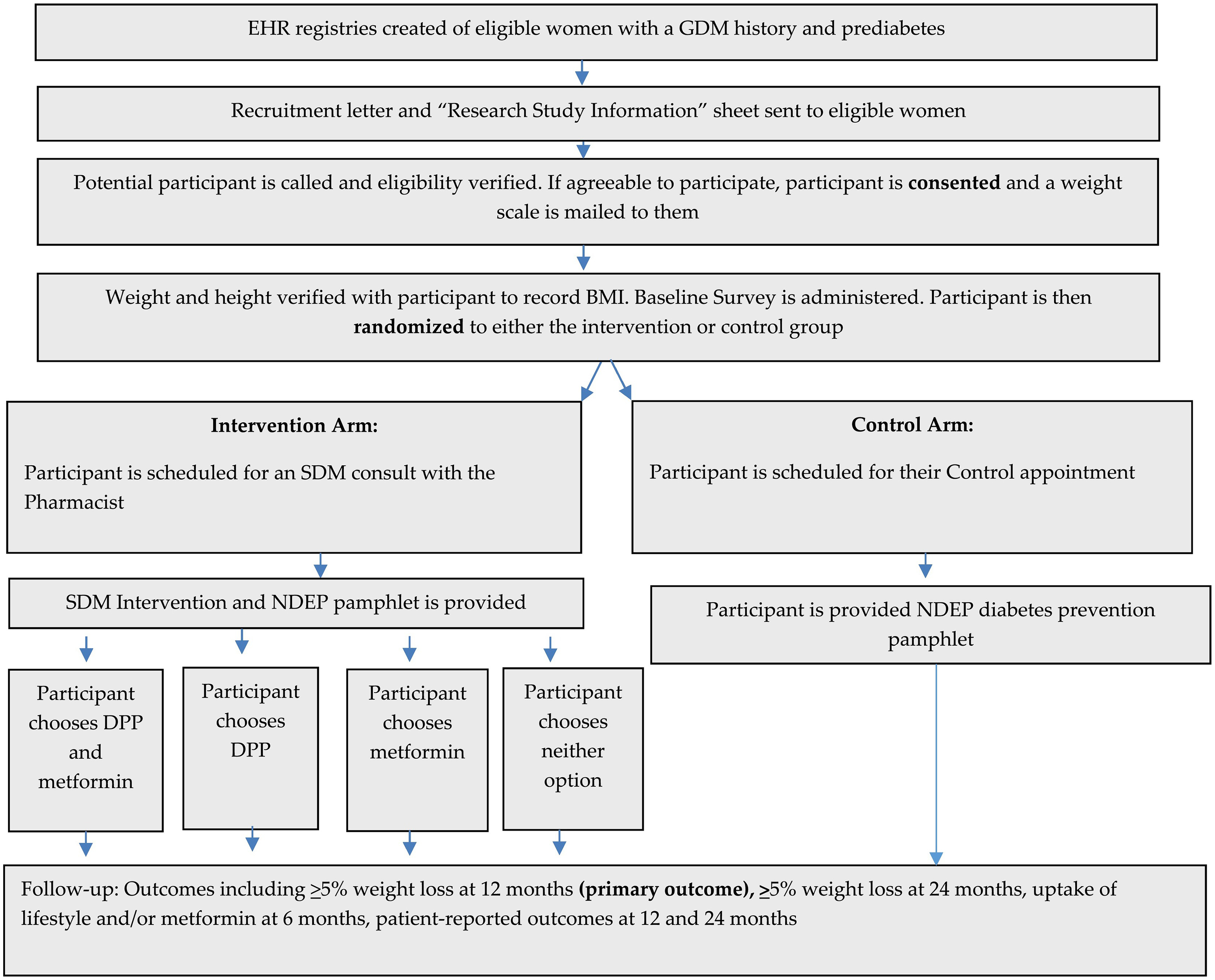
Study design.

Women who meet eligibility criteria based on our screening of electronic health record (EHR) data will receive an invitation letter. We will replicate our successful recruitment approach from prior work in SDM for diabetes prevention [36–38], first confirming patient eligibility with their physician, followed by outgoing patient intake calls, a brief touch-base call with a study principal investigator (PI), and virtual informed consent. All participants will receive a weight scale, which will be used for measuring their weight at baseline and at each follow-up. They will be instructed to weigh themselves without shoes and with emptied pockets. We use a block randomization approach stratified by health system 1:1 to the intervention versus control arm (Fig. 2). Participants will be scheduled for a face-to-face or virtual visit based on their preference, where baseline weight and survey responses will be recorded, and where, for those in the intervention arm, SDM will be performed (Fig. 2. Participants will not be blinded to the study assignment, however the lead investigators and individuals collecting follow-up data (e.g. surveys and EHR reviews) will. We will track recruitment on a monthly basis, including the numbers of patients contacted, enrolled, and randomized (Fig. 2).

### 2.5. Intervention

#### 2.5.1. Intervention Arm: protocol for SDM and implementation of patient’s decision

**Fig. 3.**
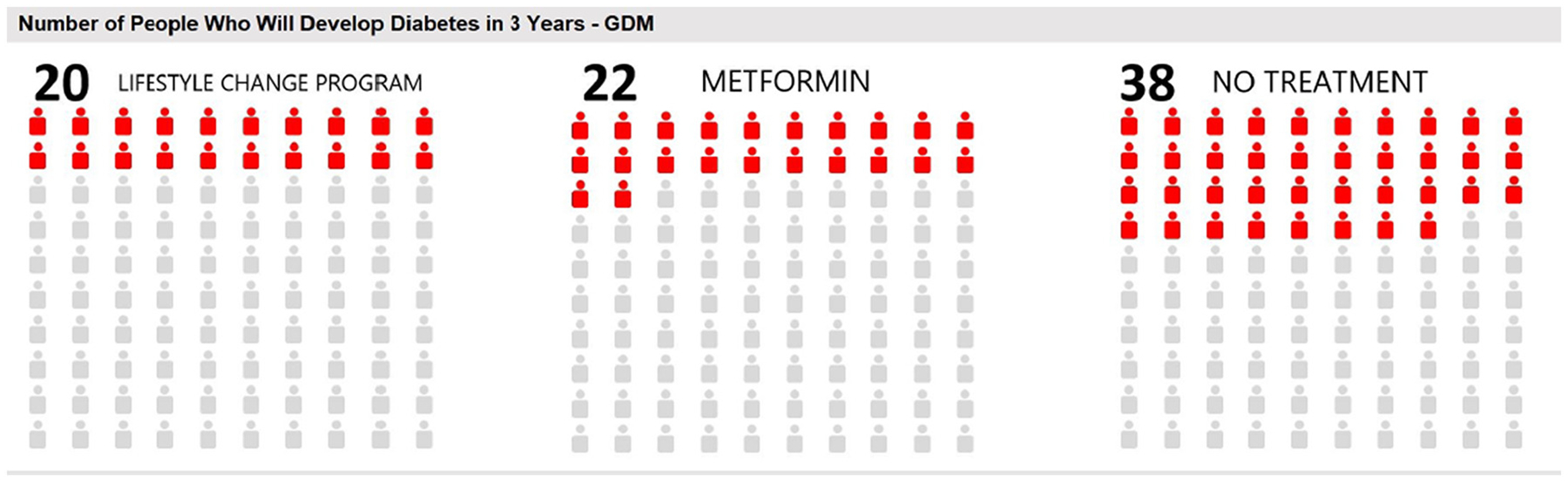
Decision Aid 100-Person Risk Chart for Incident T2DM.

Pharmacists or registered nurses trained in SDM will meet face-to-face in a clinical setting or virtually over video with each participant. During the SDM visit, the pharmacist or registered nurse will help the participant move through the steps of the decision process, using the prediabetes DA from *Healthwise*^®^, the health education nonprofit with whom we contracted. The clinician will present a 100-person chart based on data from the DPP trial which demonstrates the relative benefits of metformin and ILI in the form of a DPP for T2DM prevention among women with a history of GDM (Fig. 3). While DPP and metformin were not tested in combination, women will have the option of choosing DPP, metformin, both options, or taking no action. Participants will also receive handouts on diabetes prevention from the National Diabetes Education Program (NDEP).

After the initial SDM visit, the pharmacist or registered nurse will communicate the participant’s choice of diabetes prevention strategy to their designated primary provider, through a note in the EHR. If the participant chooses DPP, they will receive a list of in-person or online providers registered with the CDC Diabetes Prevention Recognition Program, including the DPP providers most likely to be covered by their insurance. The research team will follow up in 2–3 weeks with each participant who chose lifestyle change to help them navigate initial DPP enrollment. If a CDC-recognized DPP is not covered by a participant’s insurance, they will be provided with self-pay DPP options to enroll in if they are amenable. These self-pay DPP options vary in cost, averaging $290 for the 12-month lifestyle change program. If the participant chooses metformin, pharmacists can prescribe it with the approval of the patient’s physician, and registered nurses will propose the prescription request to the patient’s physician. The goal/prescribed dose will be 2000 mg per day of immediate release metformin. Although 850 mg twice daily of immediate release metformin was used in the original DPP trial, 1000 mg twice daily is a more commonly used dose in clinical practice and more aligned with our pragmatic approach. The UCLA pharmacists and Intermountain registered nurses will also follow up with patients who chose metformin in 2–3 weeks to assess any side effects, which they can help the patient manage by temporarily reducing the metformin dose or using other effective mitigation strategies, such as switching to 1500 mg per day of extended release metformin. For those who cannot tolerate the goal metformin dosage, we will encourage patients to take the maximum tolerated dose.

#### 2.5.2. Control Arm: in-person or virtual visit for receipt of diabetes prevention materials and weight measurement

Participants randomized to the control arm will receive an in-person or virtual visit with a research study team member, who will review the NDEP print materials with the patient and measure their weight. The same amount of time is spent with patients in the control and intervention group. Though we describe the control group as receiving “usual care,” their receipt of diabetes prevention materials and meeting with a research study team member is actually above what is typically considered usual care, since they are also presumably receiving prediabetes counseling from the PCP or physician who ordered the labs that resulted in a prediabetes diagnosis. It is primarily the responsibility of each participant’s ordering clinician to discuss prediabetes management (including lifestyle change and/or metformin) with their patient. Our study team’s interactions with the control group are meant to supplement that information.

#### 2.5.3. Training

We will use approaches recommended by the NIH Behavior Change Consortium to ensure that the SDM intervention is being delivered and received as intended [39]. These include the training of all pharmacists and registered nurses about DPP findings and SDM, which has already taken place as part of the broader diabetes prevention implementation study. We will schedule additional training modules specific to GDM that include role-playing scenarios. Our physicians will lead didactics on GDM including the pathophysiology, diagnostic criteria, and treatment of GDM during pregnancy as well as recommended post-partum care. The pharmacists and registered nurses will use an electronic template that becomes a note in the EHR for every patient visit. This template includes check boxes for each step in the structured decision-making framework.

#### 2.5.4. Financial incentives

We will provide each participant with a $35 gift card after their baseline visit, a $15 gift card after their 6-month telephone follow-up, and $35 gift cards after their 12 month and 24 month follow ups.

### 2.6. Data collection and management

We will collect data on individual participant-level outcomes and measure implementation-related costs to conduct a cost analysis of the program.

#### 2.6.1. Individual-level outcomes

We will collect data at baseline, 6 months (by telephone), 12 months, and 24 months (a total of 3 in-person or virtual visits, plus a telephone visit) for participants in both the intervention and control arms of the evaluation. A baseline questionnaire was developed to ask participants about their risk awareness of T2DM and experience with preventative measures, as well as to assess patient activation and lifestyle choices around nutrition/physical activity. At the initial visit, the intervention participants will meet with pharmacists or registered nurses while the control participants will meet with research assistants. At 6 months, all participants will receive a telephone call from a research assistant to assess uptake of lifestyle change (yes/no) and/or uptake of metformin (yes/no). Attendance of a minimum of 9 of 16 weekly lifestyle change sessions will be the threshold for uptake of lifestyle change for both groups, as this reflects the CDC’s standard for DPP recognition from 2011 to 2020. Since some patients will not be able to tolerate metformin at the goal total daily dose of 2000 mg, we will encourage patients to take the maximum dose that they can tolerate and will count any metformin dose toward the yes/no outcome of “taking metformin.” We will backfill with EHR data on medication reconciliation as needed, since nurses and medical assistants at both systems verify and document current medications at each ambulatory and inpatient visit. At the 12-month and 24-month follow-ups, all participants will conduct an in-person or virtual follow-up visit with research assistants for weight measurement and follow-up surveys. The primary outcome (proportion of participants with ≥5% weight loss at 12 months) will be determined from weights recorded at study-related visits, but we will backfill missing weights from the EHR. We will include a variable in our analysis indicating whether weight was measured based on the patient’s home scale provided by the study or backfilled from the EHR. Additional outcomes are shown in Table 1.

### 2.7. Measures and outcomes

#### 2.7.1. Specific Aim 1 – Primary outcome

Hypothesis: The intervention will result in a greater proportion of participants achieving ≥ 5% weight loss at 12 months as compared to patients receiving usual care.

For the primary analysis, we will use logistic regression to compare the proportion of participants with ≥5% weight loss between the two study arms at 12 months. The model will include indicator variables for study arm, SDM provider type (UCLA pharmacist vs. Intermountain registered nurse), and pre-selected baseline patient characteristics such as age and race/ethnicity. Given potential differences in intervention effectiveness across sites, we will conduct preliminary analyses within each site. Effect estimates for the intervention and relevant baseline covariates, if any, from the two models will be compared. Those with relatively large differences indicate potential interaction effects with site (i.e., site is an effect modifier). We will not draw final conclusions from these preliminary analyses, but in the formal analysis which combines data from the two sites, we can test interaction effects between site and intervention, and between site and other covariates as indicated by the preliminary analyses.

We used data from prior work in SDM for diabetes prevention to estimate the effect size of the proposed intervention [36–38], in which 31% of intervention participants and 13% of control participants lose ≥5% body weight at 12 months. Assuming 20% attrition, we will need at least 152 participants per arm to provide 90% power using a two-sided exact test at a significance level of 0.05. For this individually-randomized two-site study, we determined that it was not necessary to consider the intraclass correlation in the power calculation if we include a dummy variable for site (UCLA vs. Intermountain) in our analyses. We will set our enrollment goal at 310 participants (divided between 155 participants at both UCLA and Intermountain).

#### 2.7.2. Specific Aim 2 – Secondary outcomes:

Hypotheses: The intervention will result in: 1) a greater proportion of participants achieving ≥ 5% weight loss at 24 months, 2) increased uptake of lifestyle change and/or metformin at 6 months, 3) better patient-reported outcomes (physical activity, eating patterns, patient activation, health-related quality of life), 4) annual follow-up screening for progression to T2DM.

We will use the same analytic plan as described above for the primary outcome to compare intervention and usual care in terms of the rates of ≥5% weight loss at 24 months, uptake of lifestyle change and/or metformin use at 6 months, and rates of screening for T2DM. Linear regression will be used to examine patient-reported outcomes at 6, 12, and 24 months, and we will be able to measure whether patients attended a minimum of 9 of 16 weekly lifestyle change sessions even if there was a delay in enrollment. Annual follow-up screening for progression to T2DM will include any ADA-recommended screening test for the detection of T2DM, including measuring fasting plasma glucose or HbA_1c_ level or an oral glucose tolerance test. The model will include the same set of predictors outlined above for the logistic regression and also adjust for baseline patient-reported outcomes.

#### 2.7.3. Specific Aim 2 – Exploratory outcome

Hypothesis: The intervention will result in lower rates of GDM in a subsequent pregnancy.

We will use the same analytic plan as described above for the primary outcome to compare the rates of GDM in a subsequent pregnancy. GDM diagnosis will be based on ICD10 codes or positive 1-step or 2-step oral glucose tolerance test. The analysis will be restricted to participants who are not pregnant at baseline or at the 6 month follow up and who are no >3 months pregnant as the 12 month follow up. This analysis will be adjusted for baseline covariates that show differential distributions between the two groups. However, this will be an exploratory outcome, and our study is not powered to assess its significance.

#### 2.7.4. Specific Aim 3 – Cost analyses

We will not test a hypothesis with this aim, but will rather conduct a descriptive analysis of costs related to implementation of the intervention [45]. We will express results as the mean implementation cost to the healthcare system per patient who loses 5% of their body weight.

### 2.8. Statistical considerations

#### 2.8.1. Analysis plan

Study data will be examined and summarized by descriptive statistics including median, mean, and standard deviation for quantitative variables and by frequencies and proportions for categorical ones. All variables of interest will be summarized for the whole sample, by clinic sites, and by study arms. We will control for baseline covariates and use either logistic or linear regression models, depending on the type of outcome variable. Graphic displays, such as boxplots and histograms, will be used to inspect skewness and normality for quantitative variables and to identify possible outliers. All randomized subjects will be included and the intention to treat principle will be applied.

Missing data will be handled using multiple imputation techniques that assume data are missing at random [46]. Analysis will be done using the complete, imputed datasets and then combined to generate the final result. If strong evidence suggests that missing data due to loss to follow-up may be nonrandom and informative, we can perform alternative statistical modeling approaches to incorporate modeling of the dropout process. We will utilize joint models for outcomes of interest and event times (e.g., time to study dropout) in order to adjust for the possibility of non-ignorable missing data [47].

### 2.9. Safety and monitoring

Potential adverse events include musculoskeletal injuries as a consequence of supervised physical activity, lactic acidosis, and loss of confidentiality of prediabetes diagnosis due to a data breach. Every adverse event will be documented, including events that are reported to the study team and/or research associates through means other than those described above. A report will be generated for each event, including a description of the event, when and how it was reported, as well as any official chart records or documentation to corroborate the event and determine its attribution. Any serious adverse events as described above will be reported within 7 days to the outside Data and Safety Monitoring Plan monitor, and to the UCLA or Intermountain IRB as well as the National Institute of Diabetes and Digestive and Kidney Diseases (NIDDK) program officer. Additionally, safety reports will be submitted to our Data and Safety Monitor every 6 months throughout the study period. Any action resulting in a temporary or permanent suspension of this study (e.g., IRB actions) will be immediately reported to the appropriate NIDDK program official.

Institutional Review Board (IRB) approval has been obtained from the UCLA Office of Human Research Protection Program (approval number 20–001558) and from the Intermountain Healthcare IRB (approval number 1051571). This trial is registered at ClinicalTrials.gov (registration number NCT03766256, date of registration 6 December 2018).

## 3. Discussion

This study protocol describes the planned methodology of the Gestational diabetes Risk Attenuation for New Diabetes (GRAND) study, a multi-center RCT that evaluates whether SDM can help women with a history of GDM and prediabetes increase weight loss and adoption of evidence-based strategies to lower their risk of incident diabetes. We will compare the proportion of participants (intervention vs. control) achieving ≥5% weight loss and taking up lifestyle change and/or metformin in a real-world setting. We will also assess whether SDM may result in lower rates of GDM in a subsequent pregnancy and estimate the costs related to implementing SDM intervention to help healthcare practices assess the resources they will need to implement a similar program.

Given the increased lifetime risk of incident T2DM in women with a GDM history, engaging this population in evidence-based strategies for diabetes prevention is critical. This study presents a unique opportunity to address a vital area of unmet need in diabetes prevention for women with history of GDM, leveraging our health system infrastructure and our collaborative, multidisciplinary team with a strong record of accomplishment in diabetes prevention.

## 4. Conclusions

This study addresses a major unmet need and would be the first to evaluate SDM for diabetes prevention for women with a history of GDM. Our results will provide critical evidence as to whether close collaboration between patients and healthcare providers in a real-world clinical setting helps women with a GDM history make informed, timely, and effective decisions to reduce their risk of T2DM. This work will provide pragmatic, effective and sustainable approaches to increase evidence-based diabetes prevention strategies for women with a history of GDM that can be readily adopted in other health systems.

## Figures and Tables

**Table 1:** Study outcomes.

Outcomes	Measurement	When Measured	Source
Weight change, threshold (≥5% weight loss)	Weight in lbs.	Baseline, 12 m (primary outcome), 24m (secondary outcome)	In-person or virtual visit (will backfill missing data with EHR)
Uptake of DPP lifestyle program or metformin	1) Attend ine at least 9 of 16 of weekly lifestyle change sessions 2) Taking metformin (yes/no)	Baseline, 6m	DPP provider attendance records, EHR, telephone interview
Physical activity	9-item Rapid Assessment Physical Activity scale [40]	Baseline, 6m, 12m, 24 m	In-person or virtual visit and telephone interview
Eating patterns	8-item Starting the Conversation Dietary Assessment scale [41]	Baseline, 6m, 12m, 24 m	In-person or virtual visit and telephone interview
Patient activation	Altarum Consumer Engagement Measure [38,42]	Baseline, 6m, 12m, 24 m	In-person or virtual visit and telephone interview
Health-related quality of life	Short-form (SF-36) measure [43]	Baseline, 6m, 12m, 24 m	In-person or virtual visit and telephone interview
Cost per participant who loses ≥5% body weight		12 m, 24 m	EHR, payroll information, standardized cost data
Annual follow-up screening for progression to T2DM	% of women who have T2DM screening	12 m, 24 m	EHR
Rates of GDM in a subsequent pregnancy (exploratory)	% of women diagnosed with GDM during a pregnancy after enrollment	24 m	In-person or virtual visit, EHR
Other Variables			
Demographics (Age, race/ethnicity, education, etc.)		Baseline	In-person or virtual visit, EHR
Medical comorbidities		Baseline	EHR
Depression	PHQ-9 [44]	Baseline, 6m, 12m, 24 m	In-person or virtual visit and telephone interview

## Data Availability

No data was used for the research described in the article.

## Nonstandard Abbreviations

DPP: diabetes prevention program
ILI: intensive lifestyle intervention
NDEP: National Diabetes Education Program
NIDDK: National Institute of Diabetes and Digestive and Kidney Diseases
